# An Ecological Approach to Prospective and Retrospective Timing of Long Durations: A Study Involving Gamers

**DOI:** 10.1371/journal.pone.0009271

**Published:** 2010-02-17

**Authors:** Simon Tobin, Nicolas Bisson, Simon Grondin

**Affiliations:** Université Laval, Québec, Canada; Duke University, United States of America

## Abstract

To date, most studies comparing prospective and retrospective timing have failed to use long durations and tasks with a certain degree of ecological validity. The present study assessed the effect of the timing paradigm on playing video games in a “naturalistic environment” (gaming centers). In addition, as it involved gamers, it provided an opportunity to examine the effect of gaming profile on time estimation. A total of 116 participants were asked to estimate prospectively or retrospectively a video game session lasting 12, 35 or 58 minutes. The results indicate that time is perceived as longer in the prospective paradigm than in the retrospective one, although the variability of estimates is the same. Moreover, the 12-minute session was perceived as longer, proportionally, than the 35- and 58-minute sessions. The study also revealed that the number of hours participants spent playing video games per week was a significant predictor of time estimates. To account for the main findings, the differences between prospective and retrospective timing are discussed in quantitative terms using a proposed theoretical framework, which states that both paradigms use the same cognitive processes, but in different proportions. Finally, the hypothesis that gamers play more because they underestimate time is also discussed.

## Introduction

Awareness of the passing of time is a key component of time perception. Consequently, time perception studies aimed at examining this phenomenon use two different paradigms, one prospective and the other retrospective. In the prospective paradigm, participants know in advance that they will have to make a time judgment after a certain task, while in the retrospective paradigm, participants are told only afterwards that a time judgment is required [1] In both situations, time judgments are made after the task is over. However, while they execute the task, participants in the prospective condition are aware that a time judgment will be required; on the other hand, participants in the retrospective condition are informed of this additional requirement only once the task is completed. Since the time estimation in each case is made at the same moment, the key difference between the two conditions is that in the prospective paradigm, participants are aware that time is a critical component of the overall procedure, and therefore, are more likely to allow some of their attentional resources to time [2]. Generally speaking, prospective judgments about time are less variable than retrospective ones; moreover, time is perceived as longer in the prospective than in the retrospective paradigm [3]. The comparison of time judgments as a function of the time estimation paradigm is an interesting field of study as it gathers critical information about the influence of the awareness of time. It is limited, however, by two major methodological factors and a lack of ecological research.

### Methodological Obstacles

Time perception studies have been conducted far more often with the prospective paradigm than with the retrospective one [1]. Therefore, much more is known about prospective timing than retrospective timing. This stems from the fact that when study participants make retrospective time judgments, they become aware of the importance of time in the research under way, with the result that their future time judgments are prospective in nature. As a result, researchers believe that participants in prospective conditions can make numerous time estimates, while those in retrospective conditions can make only one. However, some authors suggest that numerous retrospective time estimates can be made after completing a series of tasks or activities [4]–[8].

A second, even more important factor concerns the very nature of time judgments. Not only is the number of studies on both paradigms unequal, but the possibility of comparing prospective and retrospective paradigms is limited by other variables. In fact, regardless of the paradigm under investigation, many studies have shown that the accuracy of time perception is influenced by numerous variables, such as the duration of tasks [9]–[11], their nature (empty or filled) [12], the order of presentation of stimuli [2], [8], [13], task difficulty or processing level [14], [15], event structure [4], [5], [16]–[18], expectancies [19], [20], emotions [21], [22] and body temperature [23], [24]. Since time perception experiments use different configurations of these variables, it seems useless to compare prospective studies with retrospective ones if they involve different settings (e.g., task nature and duration), as any differences observed might be caused by variables other than the time estimation paradigm itself. Therefore, comparisons of the two paradigms will be valid only if they involve studies using the same settings. Such comparisons are not abundant in the time perception literature. For example, a meta-analysis of this topic by Block and Zakay [3] reported only 16 pertinent references discussing a total of 20 valid experiments comparing prospective and retrospective judgments directly. Since this meta-analysis, very few studies involving both paradigms have been conducted [5], [8], [25]–[29]. Furthermore, these experiments, including those reported in Block and Zakay's [3] meta-analysis, led to inconsistent results: some researchers have found significant differences between the two paradigms (i.e., [12], [28], [30], [31]), while others have found none (i.e., [8], [25], [26], [32]–[34]). This has led authors like Brown [2] to conclude that too little is known about the differences between the two paradigms to provide a definitive verdict about any commonality between the processes underlying prospective and retrospective judgments.

### Unexplored Areas of Comparison

A review of experiments designed to compare both paradigms directly has highlighted another critical problem. Some areas of time perception, mainly those related to long durations or to the ecological validity of tasks, are neglected. Table 1 summarizes the durations employed by each experiment found in the literature that involves a direct comparison. The data clearly indicate that most of the durations employed are below 120 s and that long durations are left out. The fact that Block [35] qualified his 165-s duration as being a “moderately long duration” (p.148) illustrates the scope of the problem.

**Table 1:** **Durations used in each study providing a direct comparison of retrospective vs. prospective paradigms.**

Articles	Task duration
*120 s or less*	
Avni-Babad & Ritov [25]	120 s
Boltz [5]	7 s to 10 s
Bueno Martínez [54]	80 s
Brown [30]	16 s or 32 s
Gruber & Block [26]	15 s
Hicks, Miller & Kinsbourne [11]	42 s
Klapproth [27]	15 s to 45 s
Kurtz & Strube [28]	30 s or 60 s
McClain [14]	120 s
Miller, Hicks & Wilette [12]	32 s to 54 s
Predebon [31]	10 s to 50 s
Predebon [55]	48 s
Predebon [29]	12,5 s to 50 s
Zakay [37]	3 s or 6 s
Zakay [15]	12 s or 15 s
Zakay & Fallach [34]	10 s
*121 s to 20 min*	
Block [35]	165 s (exp1)
	160 s (exp2)
Block, George & Reed [56]	270 s
Brown & Stubbs [44]	14,45 min to 19,18 min (exp1)
	7,7 min to 19,6 min (exp2)
Brown & Stubbs [6]	466 s or 836 s
Kikkawa [33]	20 min
*20 min or more*	
Bakan [32]	60 min
Tobin & Grondin [8]	8 min and 24 min**

**Each participant made three consecutive tasks of 8 min, 8 min and 24 min and estimated time (prospectively or retrospectively) only at the end of all three tasks.

The review also highlighted the need to study the effect of paradigms with longer durations. To our knowledge, the only experiments that have used durations above 20 minutes in comparing both paradigms simultaneously are those of Bakan [32] and Tobin and Grondin [8], and neither of these experiments showed a significant paradigm effect on time perception. As a matter of fact, from an ecological standpoint, real life activities often exceed 20 minutes. However, ecological considerations are not the only relevant reason to study longer durations: it is theoretically sound to examine whether the distinction usually found between both paradigms still holds with much longer durations. Since attention is believed to play a major role in prospective timing, it might be asked whether attentiveness to time can be maintained at a high enough level throughout very long tasks and whether it could be the cause of the differences usually observed. Indeed, vigilance studies have shown sensitivity and performance decay over long periods (e.g.,[36]). Such decay may also apply to attention to time, which would change the expected role of attention in timing tasks.

Irrespective of paradigm comparisons, time perception experiments are mostly concerned with short durations, mainly for practical reasons. The majority of experimental designs try to carefully manipulate the attentional demands required to perform prospective and retrospective tasks. For instance, in one experiment, Hicks et al. [11] instructed participants to sort playing cards in specific ways so as to control the quantity of information they had to process. In some cases, the participants had no information to process (they were asked merely to put the cards in piles), while in others they had to process one level of information (they had to sort the cards by color) or two levels of information (they had to sort them by color and suit). The strategy was used to study intervals lasting 42 s. However, applying such a strategy to intervals lasting several hours might induce boredom. Therefore, in order to study longer durations, it might be necessary to use a more pleasant, less boring task to ensure that participants remain engaged in the task for a long period of time. From this perspective, sensitivity to the ecological value of tasks should pave the way for addressing the issue of very long durations. However, using such tasks comes with a trade-off, as it might be harder to monitor precisely the attentional demands involved.

The foregoing discussion on the type of tasks chosen for time perception research has highlighted another neglected area in the paradigm comparison literature. Indeed, most studies use non-ecological tasks, such as number searching [32], sorting cards [11], or light bulb watching [37]. However, it might be relevant to study how the difference between prospective and retrospective timing unfolds in tasks of an ecological nature, such as watching movies, playing games on the computer or browsing the Internet for several hours. Therefore, the main purpose of the present study was to examine differences between the prospective and retrospective paradigms over long durations, i.e., 12 minutes, 36 minutes and 58 minutes. Moreover, the task has more ecological validity than the tasks usually used in the time perception studies related to differences between prospective and retrospective paradigms, as it is based on a real-life situation – playing video games in gaming centers. These three long durations were selected to cover a broad range of time and assess if an increase in duration produces the same effect in both paradigms. Generally, long durations are perceived as proportionally shorter than less lengthy ones [9]. However, no study has directly examined this question using longer durations and both paradigms. Before discussing the present study and the underlying hypothesis, the problem of video game addiction will be described.

### Video Game Addiction and Time Underestimation

The use of video games as a non-temporal task provided an opportunity to study another interesting topic: the influence of video games on time perception. Indeed, a growing number of researchers and clinical psychologists are now concerned with what is called video game addiction [38]. An increasing number of video gamers show the clinical symptoms of addiction. Based on DSM-III-R criteria for addiction, Griffiths and Hunt [39] developed a questionnaire to measure addiction to video games.

Measuring the influence of this type of addiction on time perception is quite recent. Some authors suggest that gamers might underestimate time when they play, which would partly explain why they play so much [8]. In fact, some empirical data suggest that video games lead to perceived time distortions. For instance, Wood, Griffiths and Parke [40] surveyed 280 gamers about their experience of time loss while they play. Of these gamers, 82% said they often or always experience time loss when they play and 99% reported having experienced time loss at least once. This study also showed that, for many gamers, loosing track of time is one of the reasons they play video games, thus supporting the hypothesis that disrupted time perception could partially explain play time [40]. However, although this research generated convincing results when it comes to the influence of video games on time perception, it did not directly measure time estimations after game sessions. In fact, few studies have done so.

Rau, Peng and Yang [41] compared the time perception of novice and expert gamers over a 60-minute game session. According to their results, the time estimates of experts are 27% shorter than those of novice gamers, suggesting that the time perception distortion caused by video games may differ according to gamer profile. Additionally, Tobin and Grondin [8] found that a game incline profile (video game addiction, greater number of hours played per week and longer play time per game session) was associated with lower time estimates during a task involving a 24-minute video game session. Taken together, these two studies suggest a relationship between video game playing and disrupted time perception: more frequent gamers estimate time as shorter. However, the relation between time perception and video game play time cannot be described in a causal fashion. Indeed, this is a chicken-and-egg question: do gamers play a lot because they underestimate time or do they underestimate time because they play a lot? Regardless of the relationship between play time and time perception, there is a need for more empirical data that directly addresses the issue of gamers' time perception. This is why video games were chosen for the long-lasting ecological task employed in the present study.

### The Present Study

The main purpose of this study was to gather critical information about time perception by directly comparing prospective and retrospective timing using long durations (12, 35 and 58 minutes) and an ecological task (video games). Four main hypotheses were advanced in this regard. First, as is generally the case with time perception of long intervals [13], an overall underestimation of target durations was expected in both paradigms. Secondly, and along the same lines, time estimates were expected to be perceived as proportionally longer in the 12-minute condition than in the 35- and 58-minute conditions. Thirdly, as concluded by Block and Zakay [3] following their meta-analysis, prospective estimates were expected to be longer and less variable than retrospective estimates. Finally, as in the case of the Tobin and Grondin [8] study, gamer profile characteristics were expected to be a significant predictor of time perception. Since testing in an ecological environment was to be a key feature of the present study, the selected design involved testing gamers in a *network gaming center*.

## Methods

### Participants

A total of 116 people, 112 men and 4 women, recruited in two video gaming centers in Quebec City participated in the study. The mean age of participants was 22.4 years (*SD*  = 4.5 years). Participants' intended play time was the only exclusion criteria used: players who did not plan to play for at least three hours were not recruited. Participants were offered a one-hour game session as compensation for their participation. The study was approved by the *Comités d'éthique de la recherche avec des êtres humains de l'Université Laval* (CÉRUL). All subjects gave written informed consent prior to the experiment.

### Material

The study used the gaming centers' computer and network settings. Consequently, participants could play in different modes: (a) stand alone mode (i.e., participants played alone against the computer), (b) network mode (i.e., participants played with other players in the gaming center), (c) on-line mode (i.e., participants played with other players around the world) or (d) in both network and on-line modes (i.e., participants could play with friends in the gaming center and other players around the world). Also, no specific game was used in the study, as participants were instructed to play the game of their choice (see Table 2 for a complete list of the games played by each participant). In brief, by letting them play their favourite game, it was assumed that the study would involve a setting that represented an ecological environment where some gamers play video games.

**Table 2:** **List of the games played by the participants.**

Games	n
Age of Empires	7
Arma	3
Armed Army	1
Armed Assault	2
Battle For Middle Earth	2
Battlefield	27
Bioshock	2
Call of Duty	12
Civilisation 4	1
Command and Conquer	7
Company of Heroes	6
Counter-Strike	4
Everquest 2	1
Half-Life 2	1
Hellgate London	1
Linage 2×2	1
Lord of the Rings	2
Rainbow 6 Vegas	1
Serious Sam	1
Star Wars Battlefront	3
Starcraft	5
Team Fortress 2	2
Titan's Quest	1
Warcraft 3	7
World in Conflict	4
World of Warcraft	12

Three questionnaires were also completed by each participant. The first consisted of questions about time perception and control variables. In answering the time perception questions, participants had to make three time judgments in minutes and seconds. The first judgment concerned the estimated total duration (E_D_) of the game session: “Intuitively (without thinking or counting), I have the impression that this game session lasted ___ minutes and ___ seconds”. The second and third judgments consisted in estimating the minimum (Min_D_) and maximum (Max_D_) duration of their session: “I think I played for at least ____ minutes and ____ seconds and at the most ____ minutes and ____ seconds”. As for the questions relating to control variables, the aim was to gather information about variables that might influence the results: “Did you know that you needed to evaluate time?”, “Did you check the clock?”, “Did you have any time clues during your session?”, “If so, describe the clue(s)”, “Did you use the clue(s)?”

The second questionnaire consisted of questions regarding gamer profile. Variables included participants' age and sex, their level of enjoyment of the game session (on a Likert scale of 1 to 7), a comparison of their level of enjoyment of the present game session with their level of enjoyment of their usual sessions (“Compared to your usual game sessions, the present session was: less enjoyable, equally enjoyable or more enjoyable”). The questionnaire also contained a question about participants' feeling of competence in their present game session: “How would you describe your level of competence during your game session: poor, fair, good or very good?” Finally, it contained questions regarding the number of hours spent playing video games per week (none, 1–5 h, 6–10 h, 11–15 h, 16–20 h, 21–25 h or 26+ h), the name of the game the participants played and if they had previously played the game (yes/no).

The third questionnaire used was a French translation of a questionnaire developed by Griffiths and Hunt [39] to establish adolescents' addiction level. It contained the following eight questions, all requiring a “yes” or “no” response: “Do you frequently play most days?”, “Do you frequently play for long periods of time?”, “Do you play for excitement or a buzz?”, “Do you play to beat your personal high score?”, “Do you make repeated efforts to stop or decrease playing?”, “Do you become restless if you cannot play?”, “Do you play instead of attending to school related activities?”, and “Do you sacrifice social activities to play?”. According to the authors, a cut-off point of four positive answers indicates an addiction to video games.

### Procedure

Upon the players' arrival at the video game center, a research assistant asked them if they were interested in participating in a study on video games. If so, they were asked if they planned to play for at least three hours; only those who intended to play for that long were recruited for the study. Next, participants were randomly assigned to one paradigm condition (prospective or retrospective) and to one of the three duration conditions (12, 35 or 58 minutes). They were then instructed to start a game session as they normally would and were told that they would be asked questions at some point during the session. After 12, 35 or 58 minutes, the research assistant interrupted the session and asked participants to complete the three questionnaires described earlier. It is important to note that participants in the prospective condition were told before the beginning of the game session that they would be asked to make judgments about the session's duration (“At some point during your game session, we will interrupt you and ask you questions concerning the amount of time you've been playing”), whereas participants in the retrospective condition were not given this information.

## Results

The results of our study are presented in two sections, the first of which analyzes the durations estimated by the gamers, while the second deals with time estimation in the context of gamer profile.

### Time Estimates

Before presenting the results, it should be mentioned that the first three analyses were ANOVAs made on three different dependent variables: (a) the estimated-to-target duration ratio, (b) the absolute standardized error and (c) a Weber Fraction (WF)-like index. These three variables will be described and analyzed below. Also, each ANOVA was based on a 2 (paradigm: prospective vs. retrospective) ×3 (target durations: 12 vs. 35 vs. 58 minutes) inter-subject factorial design.

The estimated-to-target duration ratio (RATIO) was used to verify if there was a difference between the direction of time estimates in paradigm and target duration conditions. For this purpose, it was crucial to place the time estimates from the three target durations (12, 35 or 58 minutes) on a common basis. Thus, the RATIO was calculated by dividing the estimated duration (E_D_) by the target duration (T_D_): RATIO  =  E_D_/T_D_. Ratios higher than 1 indicated that time was perceived as longer than the target duration. According to our hypothesis, the ratios were expected to be: (a) significantly higher for the prospective paradigm, (b) under one in both paradigms and (c) significantly higher in the 12-minute condition.

**Figure 1:**
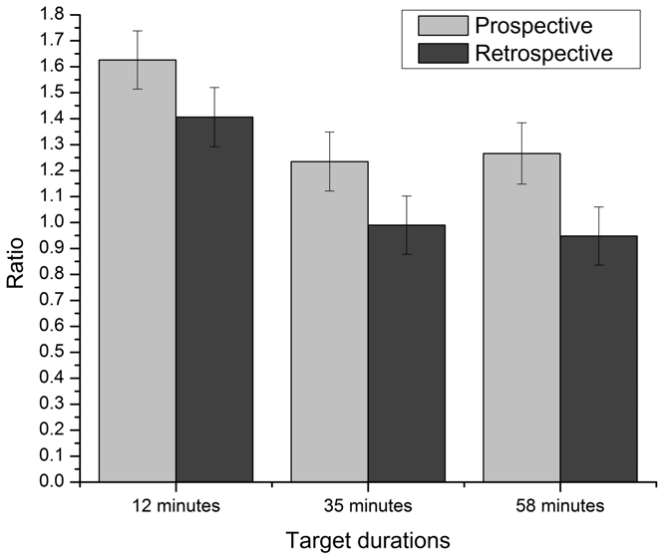
Mean estimated to target duration ratio for each target duration condition (12, 35 and 58 minutes) with each paradigm (prospective and retrospective). Bars represent standard error.

An ANOVA conducted on the RATIO revealed a paradigm main effect, *F*(1,110)  = 7.919, *p*<.05, η_p_^2^ = .067. Even though both ratios were over 1, the ratio obtained with the prospective paradigm (*M* = 1.375, *SE* = .066) was significantly higher than the one obtained in the retrospective condition (*M* = 1.115, *SE* = .065) (see Figure 1). Also, the results revealed a target duration main effect, *F*(2,110) = 8.585, *p*<.001, η_p_^2^ = .135. Pairwise comparisons revealed that RATIOs were significantly higher in the 12-minute condition than in the other conditions, but did not differ significantly between the 35- and 58-minute conditions. Finally, the interaction was not significant.

Secondly, the absolute standardized error (ASE) was used to verify if the amplitudes of the deviations of time estimates from real time differed between the paradigm and target duration conditions. This statistic is an important measure of time perception, for directional variables (like the ratio) might miss differences in the variability of estimates [30]. The absolute standardized error was calculated by putting in absolute value the difference between the time estimates and the target duration, divided by the target duration: ASE  =  absolute (E_D_−T_D_/T_D_). Greater ASE indicated that time estimates were farther from the target duration.

**Figure 2:**
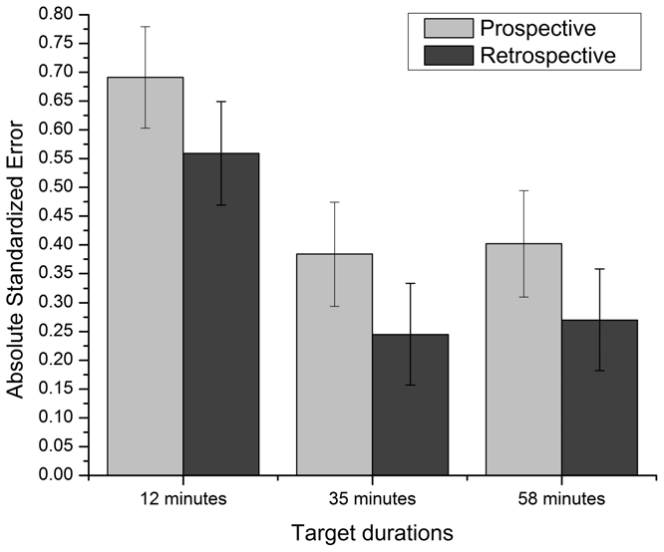
Mean absolute standardized error for each target duration condition (12, 35 and 58 minutes) with each paradigm (prospective and retrospective). Bars represent standard error.

An ANOVA conducted on the ASE revealed no significant paradigm main effect, *F*(1,110)  = 3.424, *p* = .067, η_p_^2^ = .030. Even if the difference between the paradigms was not significant, it is interesting to note that the ASE was slightly higher in the prospective paradigm (*M = *.492, *SE  = *.052) than in the retrospective one (*M = *.358, *SE  = *.051). The analysis also revealed a significant target duration main effect, *F*(2,110)  = 7.622, *p*<.05, η_p_^2^ = .122. Pairwise comparisons revealed that the ASE was significantly higher in the 12-minute condition than in the other conditions, but that there was no significant difference between the 35- and 58-minute conditions (see Figure 2). Finally, the interaction effect was not significant.

Thirdly, a WF-like index was used to determine whether the variability of time estimates differed as a function of paradigm or target duration. The WF was derived from the difference between the maximum and minimum time estimates (an estimate of variability), divided by the target duration (12, 35 or 58 minutes). Higher WF indicated higher variability in the time estimates.

**Figure 3:**
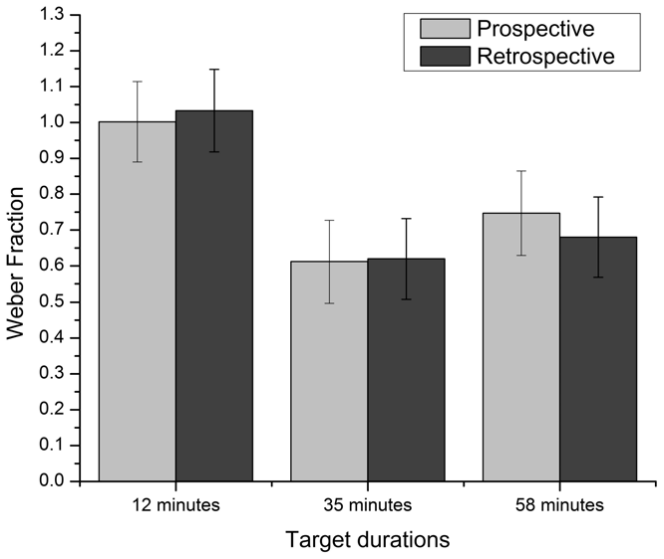
Mean Weber Fraction for each target duration condition (12, 35 and 58 minutes) with each paradigm (prospective and retrospective). Bars represent standard error.

An ANOVA completed on the WF revealed no paradigm main effect, *F*(1,110)  = .010, *p*>.05, η_p_^2^ = .000 (prospective: *M* = .787, *SE*  = .066; retrospective: *M* = .778, *SE*  = .065). However, the results revealed a target duration main effect, *F*(2,110)  = 6.858, *p*<.05, η_p_^2^ = .111. Post hoc analysis showed that the WF was significantly greater in the 12-minute condition than in the other two conditions, which did not differ significantly from each other (see Figure 3). Finally, the interaction effect was not significant.

### Gaming Profile

Stepwise regression analyses were made to verify if some gamer profile characteristics could predict the values of the three dependent variables: (a) the estimated-to-target duration ratio, (b) the ASE and (c) the WF. The following variables were included in the regressions: (a) number of hours spent playing video games per week, (b) score on the dependence questionnaire, (c) level of enjoyment during the game session, (d) a comparison of participants' level of enjoyment of the present game session with their level of enjoyment of their usual sessions, (e) their feeling of competence in the present game session, (f) the fact that they had previously played the game. Since the purpose of these regressions is to explore the predictability of the model variables at a general level, all participants have been pooled together, regardless of the paradigm and the duration conditions. Consequently, only one regression analysis was conducted for each dependent variable (Ratio, ASE and WF). Finally, the regression analysis completed on the WF is not presented here as none of the model variables were significant predictors of variance in WF.

The regression analysis conducted on the estimated-to-target duration ratio indicates that 6% (*R^2^* = .06) of the estimated-to-target duration ratio's variance can be explained by the number of hours spent playing video games per week, *F*(1,114)  = 7.513, *p*<.05. Moreover, adding the score on the dependence questionnaire to the model made it possible to explain an additional 4% (*R* ^2^ = .10) of the estimated-to-target duration ratio's variance, *F*(2,113)  = 6.225, *p*<.05. None of the other variables were retained in the model.

The regression analysis showed that 9% (*R* ^2^ = .09) of the ASE's variance can be explained by the number of hours spent playing video games per week, *F*(1,114)  = 11.038, *p*<.001. None of the other variables were retained in the model.

The number of hours spent playing video games per week seems to explain variance in both the estimated-to-target duration ratio and the ASE. Pearson correlations revealed that the number of hours spent playing video games per week was positively and significantly correlated with the estimated-to-target duration ratio (*r* = .25, *p*<.05) and with the ASE (*r* = .30, *p* = .001). In other words, the more hours participants played video games per week, the more they overestimated time and the greater the errors they made in estimating time.

## Discussion

### The Effect of Paradigm and Duration

The main goal of this study was to compare prospective and retrospective timing using long durations in order to determine if the differences between the two paradigms still hold for much longer durations. A meta-analysis conducted by Block and Zakay [3] led to the conclusion that prospective judgments are 16% higher than retrospective ones. The results of the present study show a somewhat stronger paradigm effect, with prospective estimates being on average 23% higher than retrospective ones, thus confirming the directional effect of the time estimation paradigm on the time ratio. Also, based on Block and Zakay's [3] conclusions, it was expected that prospective estimates would be more precise and less variable than retrospective ones. This was not confirmed, however, as our results showed only marginally significant differences between the impact of the paradigms on the ASE and no difference in their impact on the WF. This finding is interesting, for it shows that the difference observed in the time ratio are not caused by a larger variability or error percentage. It is only the magnitude of the overestimation, i.e., the mean estimated time that differs according to the paradigm, with the prospective paradigm leading to longer perceived durations than the retrospective one. Some experiments comparing both paradigms have revealed differences, while others have not. Nevertheless, whenever a difference has been found between the two, it has always had the same directional trend: prospective estimates are longer than retrospective ones. Indeed, no studies have found the opposite effect.

Also of interest in the present study is the target duration effect. Generally speaking, longer durations tend to be perceived as proportionally shorter than briefer ones [13]. Although this phenomenon is usually observed with very short durations (often from milliseconds to a few seconds), we proposed that the same pattern would be found for our three durations. This hypothesis was confirmed since our results showed that, proportionally, perceived time was shorter for longer durations. Indeed, the time ratio was significantly longer for the 12-minute duration (*M* = 1.516, *SE*  = .080), than for the 35- (*M* = 1.112, *SE*  = .080) and 58- (*M* = 1.107, *SE*  = .081) minute durations. The two longer durations were not significantly different, which is quite interesting. After a certain duration (35 minutes, in this case), the perceived time shortening effect seemed to stabilize. Since barely any studies have assessed time estimation for a multiple-hour range of durations, it might be expected, based on our results, that perceived time would not be much shorter after such durations. However, not only these longer durations were perceived as proportionally shorter, compared to the 12-min condition, but they also led to less inaccuracy (smaller ASE) and less variability (smaller WF). This might be explained by the use of verbal time estimates. Indeed, some studies have shown that people tend to round up to the nearest 5 minutes (for instance, a 12-min period becomes 10 minutes, and a 34-min one becomes 35 minutes) [42]. Therefore, using a 5-min error margin has a stronger effect on a smaller time scale, as in the 12-min conditions. This may explain why ASE and WF were larger in the 12-min condition. Another interesting finding is the absence of interaction between paradigm and duration, for this shows that the differences observed between both paradigms seem to hold as duration increases, thus suggesting that attentiveness to time would be maintained at a high enough level over a long period.

This experiment did not incorporate any experimental manipulations (such as different attentional or memory demands) that could explain at a cognitive level the fact that prospective estimates are longer than retrospective ones, as this was not the purpose of the study. That said, some suggestions can be made to account for this paradigm difference. Certain authors propose that prospective and retrospective timing might be based on different cognitive processes, which lead to differences in time estimates. For example, Zakay and Block [43], [44] have suggested that prospective estimates rely mainly on attention, while retrospective timing primarily involves a memory-based reconstructive process. This cognitive explanation seems plausible. However, it involves a dichotomous vision of time estimation in that it characterizes the process as prospective or retrospective. It might be useful to approach this topic from a different perspective, i.e., by seeing time perception as a continuum of attentiveness to time, as suggested by Brown and Stubbs [30], [45].

Most authors agree that the main distinction between prospective and retrospective timing is attentiveness to time [2]. However, can such attentiveness really be described in a dichotomous fashion? Several studies using prospective designs have shown that the amount or quantity of attention devoted to time increases perceived time monotonically [46]–[49]. As a matter of fact, Brown [2] reviewed the interference effect (the impact of taking attentional demands off the timing task by the use of a concurrent non-temporal task) and concluded that “these data establish the interference effect as being the most well replicated finding in all the time perception literature” (p. 119). As the attentional demands required by a non-temporal task increase, time perception shortens monotonically, suggesting that the relation between time and attentiveness to time is not best framed in a yes/no perspective, but rather in quantitative terms (How much?). Given that the level of attentiveness to time ranges from high to low in prospective timing, it might be best to view retrospective timing as being at the low end of the continuum, where attentiveness to time is very limited.

One convincing piece of evidence for this hypothesis comes from studies which have failed to find any effect of attentional demands on retrospective timing [11], [14], [15], [35], prompting the conclusion that attention is not involved in retrospective timing. This dissociation has been interpreted as strong evidence that both paradigms rely on different cognitive processes [42]. However, these studies may have used a concurrent task that involved only enough attentional demand to show an effect on prospective timing, but not on retrospective timing. Because attentiveness to time in retrospective timing is already quite low, concurrent tasks may have to be very demanding to significantly reduce the already low level of attention devoted to time [2]. Studies that have shown an interference effect in retrospective timing used a distractor task that was chosen specifically for its high difficulty level. For instance, Brown [35] used a perceptual-motor task as the concurrent task, arguing that a task like word-categorization might be too passive to produce the interference effect retrospectively. Brown's results show that the interference effect can be observed in retrospective timing as long as the concurrent task requires sufficient attentional resources. This pattern was also observed by other studies [6], [28]. As discussed earlier, the number of studies comparing both time estimation paradigms is too small to provide definitive explanations as to why these paradigms differ, even though the amplitude and direction of these differences have begun to be well described.

We suggest that seeing prospective and retrospective timing as two paradigms that differ in a quantitative, rather than a qualitative, fashion is a viable hypothesis to account for a difference such as the one observed in the present study. This could be particularly helpful for unifying models of prospective and retrospective timing (a point raised by Block and Zakay [50]) while considering the challenges to be solved by researchers working on the differences between the paradigms. Adopting a perspective in which the paradigms differ quantitatively would make it possible to develop a unified model that accounts for what is observed in both paradigms. Level of attentiveness to time might determine the relative (or proportional) use of each of the cognitive processes involved in timing, i.e., mainly attention and memory.

### Upward Shift in Time Estimates

As discussed earlier, the pattern of results observed in this study is consistent with our predictions to the effect that prospective estimates would be higher than retrospective ones and proportional perceived time would diminish as duration increased. Such findings are also consistent with the literature (Block & Zakay [3] in the case of the paradigms, and Eisler [9], to some extent, in the case of duration). However, in spite of this overall consistency, the time ratio seems to have shifted upward with long durations. This overestimation of time in an interesting task lasting as long as the one studied here is quite surprising, although the upward shift in time estimates may simply be due to one of the methodological aspects of our study. In an attempt to avoid giving participants clues about the length of the task they had to perform, we asked them during the recruitment process if they planned to spend at least 3 hours playing video games. This 3-hour span was chosen because it was much longer than the longest play time selected for the experiment, i.e., 58 minutes. Nonetheless, telling participants that the task they had to perform might last for up to 3 hours may have helped to induce an anchor effect [51], [52], for it is not totally impossible that participants judged time as being longer on the basis of this anchor. Some studies have demonstrated the influence of anchoring on time perception. When a task follows a high anchor (in this case, participants were told the experiment could last for up to three hours), time is overestimated, at least for intervals in the range of 11 minutes [1].

Another plausible explanation for the upward shift of time estimates in our study's results is the gamers' knowledge of their tendency to underestimate time. Indeed, as demonstrated by Wood et al. [40], gamers are keenly aware of the fact that they loose track of time while playing. Therefore, they might adjust their time judgments accordingly. For instance, even though they think they have played for 40 minutes, they may say that they have played for a longer period, such as 50 minutes, simply because they know that they underestimate time. This hypothesis is compelling, but little is known about the effect of previous knowledge about time distortions on subsequent time estimation. One way of studying this topic would be to try to avoid the knowledge effect by asking gamers to stop their game after a fixed duration; this would test timing resources related to time-based prospective memory (see [53]).

### Video Games

Another main goal of the present study was to gather evidence about the timing processes people use while playing video games. It was hypothesized that time perception would be significantly underestimated, thus lending support to the idea that time estimation might partially explain play time. The results were surprising and need some explanation. As mentioned earlier, the expected relation between the two variables (duration and paradigm) was observed: prospective time estimates were higher than retrospective ones and the time estimation ratio was smaller for the longer durations (35 and 58 minutes) than for the shorter one (12 minutes). Although this pattern of results was anticipated, the time ratio value indicates, for the most part, overestimation of time, which contradicts our study's hypothesis. Indeed, we expected time ratios below one, as longer durations are usually underestimated [13]. In other words, there seems to have been an upward shift toward overestimation in the time ratio values. We will first discuss these results in relation to the video game issue and then provide some suggestions as to why the results seem to have shifted upward.

Based on Tobin and Grondin [8], we thought that gamers might need a certain amount of time to “get into their game” and, therefore, that only durations that exceeded this “warm-up” period would be marked by underestimation. For the purpose of our experiment, the 12-minute duration was deemed to correspond to the warm-up period, as it was considered too short for players to fully immerse themselves in the game. Indeed, 12 minutes seems rather short compared to the usual length of a game session. For example, Grüsser et al. [38] reported that non-pathological computer gamers play 2.5 hours per day whereas pathological gamers play 4.7 hours per day. The results of the present study support the idea of an “adaptation period”, as time judgments for this period were overestimated to quite an extent. On average, gamers estimated that the 12-minute session lasted 18.1 minutes. Additional studies that exclude this adaptation period might be necessary to explore the topic of video gamers' time perception, specifically to see how these gamers perceive time once they have started playing a game in earnest.

From a video game perspective, one of the main findings of the present study is related to retrospective estimates. Even if the time ratio seems to have shifted upward in our experiment, the gamers' retrospective estimation of the longer durations (35 and 58 minutes) reflects a slight underestimation of time, thus supporting the idea that disrupted time perception could partially explain play time and the self reporting of time loss by gamers [40]. Another relevant finding is the influence of gaming profile on perceived time. Although the number of hours spent playing per week explained only a small percentage of the variance observed, it was a significant predictor of perceived time, with those who played the most making less accurate time estimates. This conclusion is consistent with other studies that have related gamer profile to perceived time [8], [41]. Therefore, this study adds some converging evidence that gaming profile is associated with somewhat different time estimation abilities. However, more studies are needed to explain the causal relation between time perception and play time: do gamers play more because they are inaccurate at estimating time or are they inaccurate because they play more?

### Concluding Comments

The main goal of this study was to compare prospective and retrospective paradigms with long durations in a natural environment. The results confirm the classical distinction found between these paradigms, with prospective estimates being longer than retrospective ones; however, the difference observed with very long intervals was greater than that usually reported. Moreover, in spite of the upward shift in our results, the 35- and 58-minute retrospective tasks showed significantly lower time estimations than the 12-minute task. Although the results don't indicate gamers underestimate time when they play, this hypothesis still receives some partial support. Indeed, a game inclined profile was associated with more inaccuracy in time estimation. Therefore, even if the results don't go in the expected direction (underestimation), this finding does support gamers might have problem with the estimation of play time (as shown by their inaccuracy).

## References

[1] Grondin S, Grondin S (2008). Methods for studying psychological time.. Psychology of time.

[2] Brown SW, Grondin S (2008). Time and attention: Review of the literature. Methods for studying psychological time.. Psychology of time.

[3] Block RA, Zakay D (1997). Prospective and retrospective duration judgments: A meta-analytic review.. Psychonomic Bulletin & Review.

[4] Boltz MG (1995). Effects of event structure on retrospective duration judgments.. Perception and Psychophysics.

[5] Boltz MG (2005). Duration judgments of naturalistic events in the auditory and visual modalities.. Perception and Psychophysics.

[6] Brown SW, Stubbs DA (1992). Attention and interference in prospective and retrospective timing.. Perception.

[7] Grondin S, Plourde M (2007). Judging multi-minute intervals retrospectively.. Quarterly Journal of Experimental Psychology.

[8] Tobin S, Grondin S (2009). Video games and the perception of very long durations by adolescents.. Computers in Human Behavior.

[9] Eisler H (1976). Experiments on Subjective Duration 1868-1975: A Collection of power function exponents.. Psychological Bulletin.

[10] Grondin S (2001). From physical time to the first and second moments of psychological time.. Psychological Bulletin.

[11] Hicks RE, Miller GW, Kinsbourne M (1976). Prospective and retrospective judgments of time as a function of amount of information processed.. American Journal of Psychology.

[12] Miller GW, Hicks RE, Willette M (1978). Effects of concurrent verbal rehearsal and temporal set upon judgments of temporal duration.. Acta Psychologica.

[13] Grondin S Eisler H, Eilser AD, Hellström A. Psychophysical issues in the study of time perception.. Psychology of time.

[14] McClain L (1983). Interval estimation: Effect of processing demands on prospective and retrospective reports.. Perception & Psychophysics.

[15] Zakay D (1993). Relative and absolute duration judgments under prospective and retrospective paradigms.. Perception & Psychophysics.

[16] Boltz MG, Macar M, Pouthas V, Friedman WJ (1992). The incidental learning and remembering of event durations.. Time, action, and cognition: Towards bridging the gap.

[17] Boltz MG (1998). The processing of temporal and nontemporal information in the remembering of event durations and musical structure.. Journal of Experimental Psychology: Human Perception and Performance.

[18] Brown SW, Boltz MG (2002). Attentional processes in time perception: Effects of mental workload and event structure.. Journal of Experimental Psychology: Human Perception and Performance.

[19] Boltz M (1989). Time judgments of musical endings: Effects of expectancies on the “filled interval effect”.. Perception and Psychophysics.

[20] Boltz MG (1993). Time estimation and expectancies.. Memory and Cognition.

[21] Bisson N, Tobin S, Grondin S (2009). Remembering the duration of joyful and sad musical excerpts.. NeuroQuantology.

[22] Droit-Volet S, Meck WH (2007). How emotions colour our perception of time.. Trends in cognitive sciences.

[23] Hancock PA (1993). Body temperature influence on time perception.. Journal of General Psychology.

[24] Wearden JH, Penton-Voak IS (1995). Feeling the heat: Body temperature and the rate of subjective time revisited.. Quarterly Journal of Experimental Psychology Section B.

[25] Avni-Babad D, Ritov I (2003). Routine and the perception of time.. Journal of Experimental Psychology: General.

[26] Gruber RP, Block RA (2003). Effect of caffeine on prospective and retrospective duration judgements.. Human Psychopharmacology: Clinical and Experimental.

[27] Klapproth F (2007). Time perception, estimation paradigm, and temporal relevance.. Perceptual and Motor Skills.

[28] Kurtz RM, Strube MJ (2003). Hypnosis, attention and time cognition.. International Journal of Clinical and Experimental Hypnosis.

[29] Predebon J (1999). Time judgments as a function of clock duration: Effects of temporal paradigm and an attention-demanding nontemporal task.. Perceptual and Motor Skills.

[30] Brown SW (1985). Time perception and attention: the effects of prospective versus retrospective paradigms and task demands on perceived duration.. Perception & Psychophysics.

[31] Predebon J (1995). Prospective and retrospective time estimates as a function of clock duration.. Perceptual & Motor Skills.

[32] Bakan P (1955). Effect of set and work speed on time estimation.. Perceptual & Motor Skills.

[33] Kikkawa M (1983). The effect of distribution of attention upon prospective and retrospective estimations of long temporal intervals.. Journal of Child Development.

[34] Zakay D, Fallach E (1984). Immediate and remote time estimation: a comparison.. Acta Psychologica.

[35] Block RA, Macar M, Pouthas V, Friedman WJ (1992). Prospective and retrospective duration judgment: the role of information processing and memory.. Time, action, and cognition: Towards bridging the gap.

[36] See JE, Howe SR, Warm, JS, Dember WN (1995). Meta-analysis of the sensitivity decrement in vigilance.. Psychological Bulletin.

[37] Zakay D (1992). The role of attention in children's time perception.. Journal of Experimental Child Psychology.

[38] Grüsser SM, Thalemann R, Griffiths MD (2007). Excessive computer game playing: evidence for addiction and aggression?. Cyberpsychology & Behavior.

[39] Griffiths MD, Hunt N (1998). Dependence on computer games by adolescents.. Psychological Reports.

[40] Wood RTA, Griffiths MD, Parke A (2007). Experience of time loss among videogame players: An empirical study.. Cyberpsychology & Behavior.

[41] Rau P-LP, Peng S-Y, Yang C-C (2006). Time distortion for expert and novice online game players.. Cyberpsychology and Behavior.

[42] Yarmey DA (2000). Retrospective duration estimations for variant and invariant events in field situations.. Applied Cognitive Psychology.

[43] Zakay D, Block RA (1997). Temporal cognition.. Current Direction in Psychological Science.

[44] Zakay D, Block RA (2004). Prospective and retrospective duration judgments: an executive-control perspective.. Acta Neurobiologiae Experimentalis.

[45] Brown SW, Stubbs DA (1988). The psychophysics of retrospective and prospective duration timing.. Perception.

[46] Casini L, Macar F (1997). Effects of attention manipulation on judgments of duration and on intensity in the visual modality.. Memory and Cognition.

[47] Casini L, Macar F, Grondin S, Macar M, Pouthas V, Friedman WJ (1992). Time estimation and attentionnal sharing.. Time, action, and cognition: Towards bridging the gap.

[48] Grondin S, Macar F, Macar M, Pouthas V, Friedman WJ (1992). Dividing attention between temporal and nontemporal tasks: A performance operating characteristic—POC—analysis.. Time, action, and cognition: Towards bridging the gap.

[49] Macar F, Grondin S, Casini L (1994). Controlled attention sharing influences time estimation.. Memory and Cognition.

[50] Block RA, Zakay D, Hoerl C, McCormack T (2001). Retrospective and prospective timing: memory, attention and consciousness.. Time and memory: Issues in philosophy and memory.

[51] Tversky A, Kahneman D (1974). Judgment under uncertainty Heuristics and biases.. Science.

[52] Thomas KE, Handley SJ (2008). Anchoring in time estimation.. Acta Psychologica.

[53] Graf P, Grondin S, Glicksohn J, Myslobodsky MS (2006). Time Perception in Time-Based Prospective Memory.. Timing the Future: The Case for a Time-Based Prospective Memory.

[54] Bueno Martínez MB (1990). Efectos de los cambios cognitivos y del esfuerzo de procesamiento sobre los juicios de duración prospectivos y retrospectivos. [Effects of cognitive changes and processing effort on judgment of prospective and retrospective duration].. Estudios de Psicología.

[55] Predebon J (1996). The effects of active and passive processing of intervals events on prospective and retrospective time estimates.. Acta Psychologica.

[56] Block RA, George EJ, Reed MA (1980). A watched pot sometimes boils: A study of duration experience.. Acta Psychologica.

